# Recurrent pseudohypopyon in association with primary vitreoretinal lymphoma: a case report

**DOI:** 10.1186/s12886-016-0279-0

**Published:** 2016-07-08

**Authors:** Masahiro Kitao, Noriyasu Hashida, Kohji Nishida

**Affiliations:** Department of Ophthalmology, Osaka University Graduate School of Medicine, E7, 2-2 Yamadaoka, Suita, Osaka 565-0871 Japan

**Keywords:** Primary vitreoretinal lymphoma, Recurrence, Pseudohypopyon, Methotrexate

## Abstract

**Background:**

Primary vitreoretinal lymphoma (PVRL) is a rare and fatal ocular malignancy that is mostly classified as diffuse large B cell lymphoma (DLBCL). PVRL is often fatal because of its association with the central nervous system (CNS). PVRL frequently masquerades as uveitis and sometimes recurs in clinical findings as keratic precipitates (KPs) and subretinal lesions. Pseudohypopyon is one of the clinical findings of the local recurrence of PVRL and is treated with radiotherapy; however, the effectiveness of local control with an intravitreal injection of methotrexate (MTX) has not yet been determined. We herein present a case of recurrent vitreoretinal lymphoma that repeatedly developed pseudohypopyon and treated with intravitreal MTX.

**Case presentation:**

A 64-year-old woman was diagnosed with PVRL involving primary central nervous system lymphoma (PCNSL). She received radiotherapy to the whole brain and a local ocular treatment, which resulted in temporary remission of the disease. Pseudohypopyon was detected during the follow-up. It was 2-mm high, viscous, and swollen in the center. Anterior chamber biopsy revealed the presence of atypical lymphocytes, indicating the recurrence of DLBCL. Pseudohypopyon was treated with intravitreal methotrexate and completely disappeared. Pseudohypopyon has since repeatedly appeared and been treated with intravitreal MTX each time. The recurrence of PVRL with KPs and subretinal invasion was treated with intravitreal MTX each time. Recurrence with pseudohypopyon was not simultaneous with KPs or subretinal invasion. No CNS involvement was detected during the observation period.

**Conclusions:**

Pseudohypopyon is one of the signs of recurrent vitreoretinal lymphoma. Although pseudohypopyon was temporarily controlled with intravitreal MTX, this treatment did not completely induce its remission.

## Background

Primary vitreoretinal lymphoma (PVRL) is a subset of primary central nervous system lymphoma (PCNSL), mostly diffuse large B-cell lymphoma (DLBCL). PVRL is often fatal because of its association with the CNS [1]. Approximately 80 % of PVRL patients eventually develop PCNSL, and the 5-year survival rate of PVRL has been reported to be 30–60 % [2]. PVRL frequently masquerades as uveitis and difficulties are associated with making an accurate diagnosis in the absence of CNS involvement. PVRL is sometimes misdiagnosed as uveitis because the clinical features of PVRL masquerade as uveitis and most PVRL cases are initially responsive to corticosteroids. Patients with PVRL generally have blurred vision and floaters. The clinical features of PVRL are keratic precipitates (KPs), vitreous opacities, and subretinal invasion.

PVRL often recurs even though it is treated systemically. In recurrent cases, the same ocular findings, such as KPs, vitreous cells, and subretinal invasion, are observed [3]. Pseudohypopyon is rare, but one of the significant clinical manifestations of local recurrence. A previous study reported that pseudohypopyon was observed in patients with a malignant tumor and Ewing’s sarcoma [4]. The standard treatment for patients with PVRL is a combination of systemic chemotherapy and radiation [2]. Intravitreal methotrexate (MTX) and intravitreal rituximab are currently the standard treatment options for intraocular lesions [1]. The effectiveness of high-dose (HD) MTX for the treatment of PVRL with CNS involvement has also been reported [3]. The same strategy has been selected to treat recurrent cases, depending on patient systemic conditions [5]. There are several case reports in the literature on the treatment of PVRL that relapsed with pseudohypopyon. These findings suggest that it is possible to treat and control pseudohypopyon in recurrent PVRL with local radiotherapy [3]. However, local chemotherapy and its effectiveness have not yet been examined in these patients.

In the present study, we reported the rare complication of recurrent pseudohypopyon in a patient with PVRL treated with intravitreal MTX. We also monitored treatment-associated changes in the lesion by serial examinations.

## Case presentation

A 64-year-old woman presented to a local doctor in 2000 with blurred vision in her right eye. She was diagnosed with chronic iridocyclitis and treated with topical corticosteroids with limited improvements. More detailed examinations were not performed at that time. In 2003, she consulted a neurologist for depression, and thereafter was diagnosed with CNS lymphoma by brain MRI. On presentation, best-corrected visual acuity (BCVA) was 20/20 in the right eye and 20/20 in the left eye, and intraocular pressure was normal. A slit-lamp examination showed KPs and inflammatory cells (2+) in the anterior chamber of the right eye. A fundus examination of the right eye revealed vitreous opacities (3+). Slit-lamp and fundus examinations of the left eye showed no abnormalities. Since the identification of lymphoma cells in the vitreous is required for a diagnosis of PVRL, we performed diagnostic vitrectomy. A vitreous biopsy sample indicated increased IL-10 levels (379 ng/mL) and an elevated IL-10 to IL-6 (12.6 pg/mL) ratio. A cytological analysis also showed large atypical lymphoid cells, resulting in DLBCL. She was diagnosed with PVRL with CNS involvement and started treatment. She underwent one course of intravenous HD MTX chemotherapy (3.5 g/m^2^) and radiotherapy to the right eye of 40Gy. Brain recurrence has not been observed since then.

**Fig. 1:**
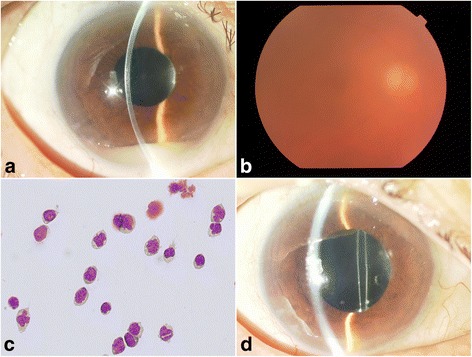
Clinical ocular findings before the treatment. **a** Slit-lamp examination of pseudohypopyon before intravitreal methotrexate (MTX) showing white and viscous accumulation of cells. **b** Fundus photograph showing strong vitreous opacities. **c** A cytological analysis revealed large atypical lymphoid cells, resulting in DLBCL. **d** Pseudohypopyon had completely disappeared 1 week following treatment

Bilateral involvement was detected during the observation period. KPs and anterior chamber cells are also observed in the left eye. She had been repeatedly treated with a dose of 400 μg of intravitreal MTX and a dose of 1 mg of intravitreal rituximab for recurrent intraocular lesions between 2004 and 2011: intravitreal MTX; 7 times for the right eye/3 times for the left eye: intravitreal rituximab; 7 times for the right eye. The MTX injections were administered using a 30-gauge needle after application of a topical anesthesia and 5 % povidone iodine disinfection in the superior temporal quadrant 3.5 mm posterior to the limbus. Intravitreal rituximab was also administered to the recurrence from 2008 to 2009 and was temporally effective; however, the iridocyclitis as the side effects of injection was developed each time. Therefore, intravitreal rituximab was discontinued. In 2012, she presented at our hospital with blurred vision in her right eye. BCVA in her right eye was 20/250. A slit-lamp examination of the right eye showed a 2-mm high viscous pseudohypopyon, anterior chamber cells (3+), and flare (2+) (Fig. 1a). KPs were not observed. A fundus examination of the right eye showed (3+) strong vitreous opacities (Fig. 1b); however, subretinal invasion was not observed. There was no abnormal finding in her left eye at the time of right eye recurrence. Anterior chamber biopsy revealed the presence of atypical lymphocytes, indicating the recurrence of DLBCL (Fig. 1c). Intravitreal MTX was initiated. One week later, pseudohypopyon completely disappeared, while anterior chamber cells (1+) remained (Fig. 1d). Vitreous opacities also disappeared. Symptoms resolved completely within a few months by the sequential treatments.

**Fig. 2:**
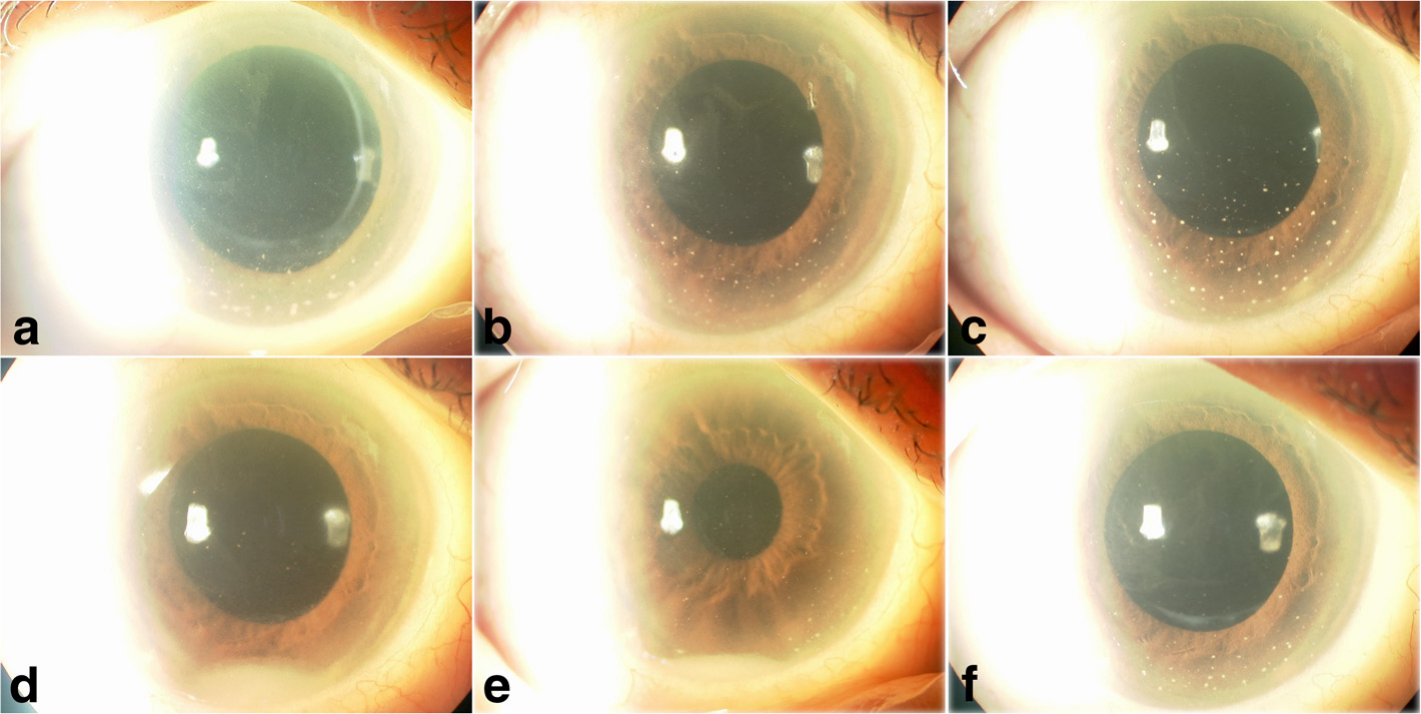
Slit-lamp photograph showing representative clinical manifestation of the local anterior segment recurrence of PVRL. PVRL recurrence was observed at **a** 7, **b** 9, and **c** 13 months with KPs, **d** 14 and **e** 15 months with pseudohypopyon, and **f** 23 months with KPs. Intravitreal injection of methotrexate temporally controlled each local recurrence

**Fig. 3:**
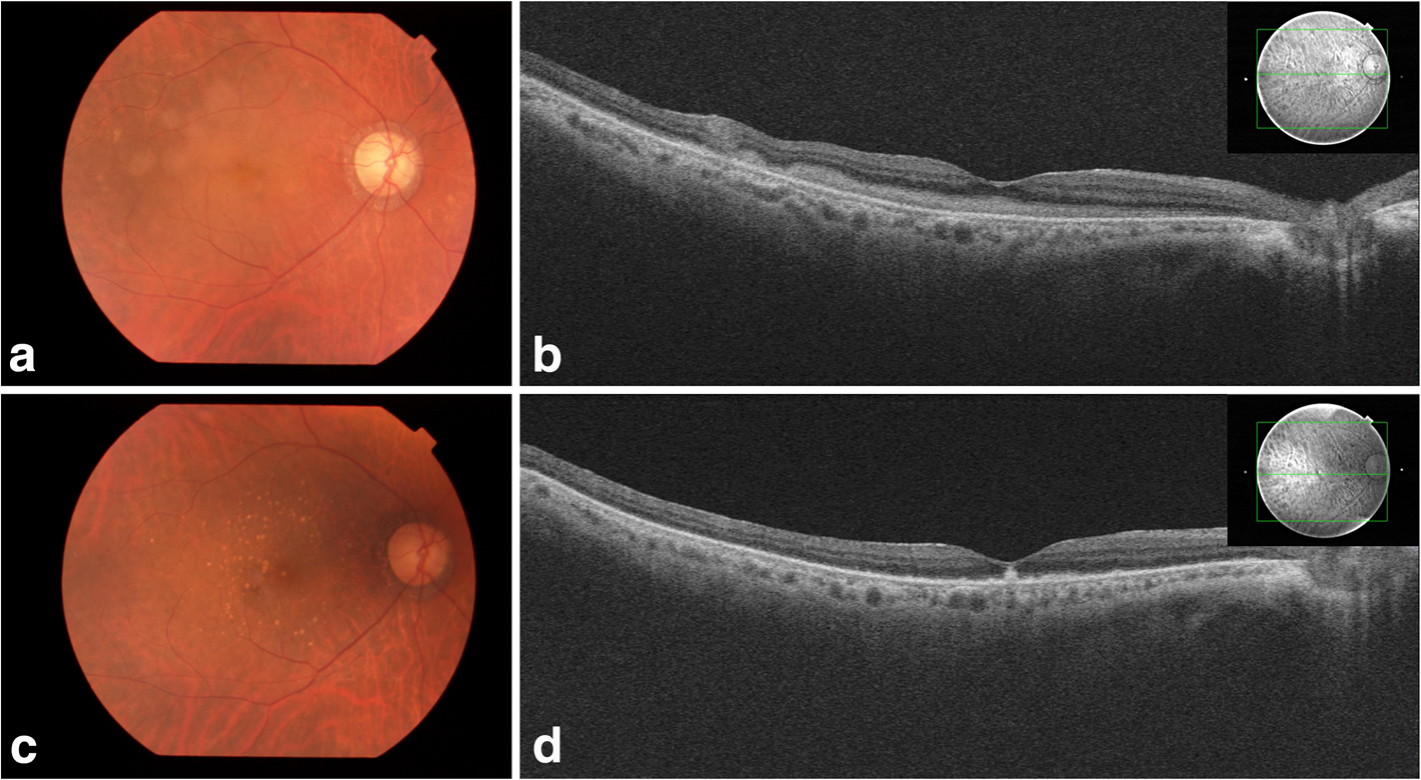
PVRL recurrences with subretinal invasion before (**a**, **b**) and after (**c**, **d**) the treatment

The recurrence of PVRL with pseudohypopyon appeared 14, 15, and 18 months after the first appearance of pseudohypopyon (Fig. 2d, e). An intravitreal MTX was indicated for the management of each recurrence, resulting in the resolution of symptoms. Moreover, the recurrence of PVRL with KPs (Fig. 2a–c, f) and subretinal invasion (Fig. 3a, b) were also noted (5 times for KPs/1 time for subretinal invasion). Intravitreal MTX was also an effective treatment for recurrence (Fig. 3c, d). Recurrence with pseudohypopyon was not simultaneously observed with KPs, vitreous opacities, or subretinal invasion. In May 2015, there had been no recurrence for 6 months. Visual acuity remained at 20/50 in her right eye and 20/20 in her left eye. The subsequent clinical course of left eye was uneventful. No CNS involvement was noted during the observation period.

## Discussion

PVRL typically responds well to initial treatments; however, relapse rates are high and repeated treatments are sometimes needed. There are various types of clinical features for the recurrence of PVRL. Pseudohypopyon is relatively rare and its pathogenesis is not fully understood. Previous studies reported that pseudohypopyon was treated by a combination of systemic chemotherapy and radiation [6, 7]; however, a treatment with intravitreal MTX has not yet been described in the literature. Therefore, to the best of our knowledge, we are the first to describe the treatment of PVRL recurrence with pseudohypopyon with intravitreal MTX.

Several studies have reported pseudohypopyon with the recurrence of PVRL. Lobo A et al. described a patient with a rare and unusual presentation of hypopyon uveitis who was eventually diagnosed with DLBCL [7]. Papaliodis GN et al. reported that a patient with a previous medical history of peripheral B-cell lymphoma developed hypopyon 3 months after R-CHOP chemotherapy and prophylactic intrathecal chemotherapy [6]. In both cases, the combination of systemic chemotherapy and radiation was effective to cause the remission of pseudohypopyon. In cytological analysis, pseudohypopyon cells are medium to large pleomorphic lymphoid cells with irregular hyperchromatic nuclei and prominent mitotic activity. The phenotypes of these cells were found to be partly positive for CD20 [6, 7]. A pathological analysis of a biopsy sample from the aqueous humor revealed the same findings.

An optimal treatment has not yet been established for recurrent PVRL. However, the general treatment for PVRL is a combination of systemic chemotherapy and radiation. An intravitreal injection of MTX and rituximab was recently shown to be effective for intraocular lesions, and has also been used in the treatment of PVRL local recurrence; however, its application to recurrent pseudohypopyon has not yet been described. In our case, we temporarily controlled the local lesion with an intravitreal injection of MTX at the time of each recurrence; however it did not completely go into remission. Comparing the effectiveness of intravitreal MTX with local radiation therapy, the severe side effects such as radiation retinopathy and keratopathy were observed [6]. In contrast, no serious side effect was observed in our case.

Regarding the pathogenesis of pseudohypopyon, previous studies reported that retinal lesions of PVRL emerge from the retinal pigment epithelium and Bruch membrane [8, 9], however; the formation of pseudohypopyon is not fully understood. Another study demonstrated the clearance of inflammatory cells in the anterior chamber using an experimental hypopyon model of SD (Sprague–Dawley) rats. According to this study, experimentally transferred cells in the anterior chamber were removed by shifting into the iris tissue, and migrating through the connective tissue close to the major arterial circle of the iris and the connective tissue surrounding blood vessels infiltrating the sclera, getting to the limbal and episcleral subconjunctival tissues [10]. Combined with these findings, it is likely that tumor cells of pseudohypopyon originate from the retina, choroid, and iris, and accumulate in the anterior chamber. No apparent retinal lesion was observed in timing of the appearance of pseudohypopyon; however, the tumor cells of pseudohypopyon may migrate from the retina and/or iris vessels or leak through the perivascular tissue. Therefore, intravitreally administered MTX may penetrate the anterior chamber and control the pathological condition, and, as such, intravitreal MTX may be effective for the control of pseudohypopyon.

## Conclusion

In conclusion, we herein report a case of pseudohypopyon as a clinical manifestation of PVRL recurrence. The pathogenesis of pseudohypopyon remains to be addressed. Although pseudohypopyon was temporarily controlled by intravitreal MTX, it did not completely induce its remission.

## Abbreviations

CNS, central nervous system; DLBCL, diffuse large B cell lymphoma; HD, high-dose; KPs, keratic precipitates; MTX, methotrexate; PCNSL, primary central nervous system lymphoma; PVRL, primary vitreoretinal lymphoma
